# The Plasticity of Th17 Cells in the Pathogenesis of Rheumatoid Arthritis

**DOI:** 10.3390/jcm6070067

**Published:** 2017-07-10

**Authors:** Shigeru Kotake, Toru Yago, Tsuyoshi Kobashigawa, Yuki Nanke

**Affiliations:** Institute of Rheumatology, Tokyo Women’s Medical University, 10-22 Kawada-cho, Shinjuku, Tokyo 162-0054, Japan; toruyago@gmail.com (T.Y.); kbsgwtys@gmail.com (T.K.); ynn@twmu.ac.jp (Y.N.)

**Keywords:** CD161, classic Th1, nonclassic Th1, plasticity, rheumatoid arthritis, Th17

## Abstract

Helper T (Th) cells play an important role in the pathogenesis of autoimmune diseases, including rheumatoid arthritis (RA). It has been revealed that Th17 cells can shift to Th1 cells (i.e., “nonclassic Th1 cells”), which are reported to be more pathogenic than Th17 cells per se. Thus, the association of Th cells in the pathogenesis of autoimmune disease has become more complicated. We recently reported using peripheral blood from untreated and early-onset RA patients that the ratio of CD161+Th1 cells (i.e., Th17-derived Th1 cells to CD161+Th17 cells) is elevated and that levels of interferon-γ (IFNγ)+Th17 cells are inversely correlated with levels of anti-CCP antibodies. Here, we review the plasticity of Th17 cells in the pathogenesis of RA, suggesting possible implications for novel therapies.

## 1. Introduction

**Figure 1:**
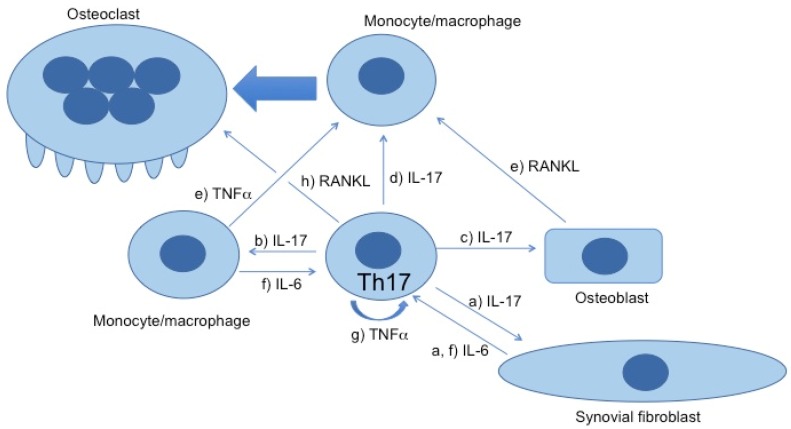
The role of Th17 cells in the pathogenesis of rheumatoid arthritis (RA). Th17 cells play a central role in the pathogenesis: (**a**) IL-17 stimulates synovial fibroblasts to produce IL-6 [5] and (**b**) macrophages to produce TNFα [6]; (**c**) IL-17 also stimulates osteoblasts to produce RANKL, potently inducing osteoclastogenesis [2,3]; (**d**) In addition, IL-17 induces osteoclastogenesis from monocytes alone in the absence of osteoblasts or RANKL [7]; (**e**) RANKL and TNFα synergistically induce osteoclastogenesis [7]; (**f**) IL-6 induces differentiation of Th17 cells [8]; (**g**) TNFα is produced by Th17 cells per se; thus, Th17 cells differentiate to nonclassic Th1 in an autocrine or paracrine manner [9]; (**h**) RANKL expressed on the surface of Th17 cells converts nonresorptive osteoclasts to resorptive osteoclasts via cell–cell contact [10]. RANKL, receptor activator of nuclear factor ĸB.

Rheumatoid arthritis (RA) is a systemic autoimmune disease with chronic joint inflammation and destruction, and is characterized by activated T cells [1]. In 1999, we reported that IL-17 from activated human T cells in the synovial tissues of RA patients is a potent stimulator of osteoclastogenesis [2]. Th17 cells have been reported to play important roles in the pathogenesis of RA [3,4] since their identification in 2005 [5] (Figure 1). In addition, it has been revealed that Th17 produce IL-21, IL-22, TNFα, except IL-17.

Several groups have reported IL-17 as an important cytokine in the early phase or the disease-onset phase of RA. The peripheral level of IL-17 is significantly high in RA patients whose disease durations are less than 9 weeks [11]. In addition, the levels of IL-17 in individuals before RA onset is significantly higher than that in patients after RA onset [12]. In 2013, Chalan et al. reported that the number of circulating CD4+CD161+T lymphocytes are elevated in seropositive arthralgia before the onset of RA but decreased in patients with newly diagnosed RA [13]. In contrast, a regulatory variant in CC chemokine receptor 6 (CCR6, a specific marker for Th17 cells [14,15]) is related to RA susceptibility [16]. Thus, many findings support that IL-17 plays a crucial role in the disease onset or the early phase of RA.

Helper T (Th) cells play an important role in the pathogenesis of autoimmune diseases, including RA [17]. In 2005, Th17 cells were reported as a novel Th cell [5]. In addition, it was revealed that Th17 cells can shift to Th1 cells (i.e., “nonclassic Th1 cells”), which are reported to be more pathogenic than Th17 cells per se [18,19,20,21]. Thus, a novel dichotomy—classic Th1 cells and nonclassic Th1 cells—appeared [19,20]. The association of Th cells in the pathogenesis of autoimmune disease has become more complicated. In addition, numerous “Th1 cells” studies from before nonclassic Th1 cells were discovered may need to be evaluated again, because “Th1 cells” in the previous studies included both classic and nonclassic Th1 cells. We review the plasticity of Th17 cells in the pathogenesis of RA.

## 2. Plasticity of Th17

In 1986, Mosmann et al. reported the dichotomy of mouse helper T cells Th1 and Th2. Th1 cells produce interferon-γ (IFNγ), stimulating cellular immunity and protecting intracellular pathogens [22]. In contrast, Th2 cells produce IL-4, inducing humoral immunity. Based on this Th dichotomy, the “Th1/Th2 paradigm”, autoimmune diseases were divided into two categories—“Th1 diseases” and “Th2 diseases”. Rheumatoid arthritis (RA) was supposed to be a Th1 disease; however, both sensitive and specific radioimmunoassays and a standard cytopathic inhibition assay showed little or no IFNγ in synovial fluids [23,24,25]. Thus, in the 1990s, both clinical and basic researchers began to think that the “Th1/Th2 paradigm” was too simplistic.

In 1996, human IL-17 was cloned as a novel cytokine [5]. We demonstrated that IL-17 plays a crucial role in the pathogenesis of RA and osteoclastogenesis in 1999 [2]. (We call “IL-17A” “IL-17” in the current review.) In 2005, the 3rd Th cell type, Th17, was reported, which was firstly called “Th_IL-17_” [26,27]. Th17 cells play a role in protective immunity against extracellular bacterial infection [28]. We also demonstrated that IL-17 induces human osteoclastogenesis even in the absence of osteoblasts [7] and that the IL-23-IL-17 axis plays an important role in mouse arthritis and human osteoclastogenesis [29]. It has been revealed that Th17 plays an important role in the pathogenesis of many autoimmune diseases including RA [5].

In 2007, Annunziato et al. reported Th17 cells producing both IL-17 and IFNγ, coining “Th17/Th1 cells” and that Th17 cells shift to Th17/Th1 by stimulation of IL-12 [30]. In 2008, Cosmi et al. reported that CD161 is a marker of human Th17 cells and that Th17 cells are derived from CD161+ naïve T cells in human umbilical cord blood [31]. In addition, in 2011, they demonstrated that CD4+CD161+T cells can shift from Th17 to the Th17/Th1 or even Th1 phenotype in SF of patients with juvenile idiopathic arthritis (JIA) [18]. CD161+ Th1 cells, i.e., Th17-derived Th1 cells was termed “nonclassic Th1 cells” [21], showing a novel dichotomy, classic Th1 cells and nonclassic Th1 cells [19,20]. In 2016, we demonstrated the detection of IFNγ+IL-17+ cells in salivary glands of patients with Sjögren’s syndrome and Mikulicz’s disease [32]. Thus, the plasticity of human Th17 cells has been demonstrated.

Th17 cells are highly unstable and easily shift to Th1 cells [33,34]. In addition, it has recently been reported that TGFβ and IL-6 induce Th1-to-Th17-cell transdifferentiation in the mouse gut, as a “reverse plasticity” [35]. The discovery of this alternative pathway of Th17- and Th17/Th1-cell generation is likely to have important implications for both immune-mediated diseases and protective immune responses, in particular in the gut [36].

## 3. Th17-Derived Th1 Cells (CD161+ Th1 Cells, Nonclassic Th1 Cells)

In 2008, Cosmi et al. reported that CD161 (or NKR-P1A) gene is up-regulated in human Th17 clones and that all IL-17–producing cells are contained in the CD161+ fraction of CD4+ T cells present in the circulation or in inflamed tissues [31]. More importantly, they showed that all IL-17–producing cells originate from CD161+ naïve CD4+ T cells of umbilical cord blood in response to the combined activity of IL-1β and IL-23 [31]. In 2009, Kleinschek et al. identified human CD161+ CD4 T cells as a resting Th17 pool that can be activated by IL-23 and mediate destructive tissue inflammation using blood and fresh colon specimens from patients with Crohn’s disease [37]. Thus, these findings indicated that CD161 is a novel surface marker for human Th17 cells.

Two ligands have been identified for human CD161. One ligand for CD 161 was identified as the lectin-like transcript-1 (LLT-1) in 2005 [38]. It is suggested that CD161 plays an important role in favoring trans-endothelial migration of Th17 cells into tissues [31]. Another ligand of CD161 was identified as the proliferation-induced lymphocyte-associated receptor (PILAR) in 2008 [39]. In the absence of CD28 costimulation, PILAR signaling through CD161 supports T-cell proliferation by increasing the expression of antiapoptotic Bcl-xL and induces secretion of Th 1 cytokines [39]. In addition, PILAR was expressed by 10% of CD4+ T cells in synovial fluid from one patient with RA [39]. Thus, CD161 with these ligands plays important roles in both human immunity and the pathogenesis of RA.

In 2010, Nistala et al. showed that Th17/Th1 cells from the joints of children with inflammatory arthritis highly express both Th17 and Th1 lineage-specific transcription factors, receptor-related orphan receptor _C_2 (ROR_C_2) and T-bet [40]. They also showed that Th17 cells shift to Th17/Th1 with low TGFβ and high IL-12 levels, which mimics the condition of the disease site. In addition, Th17/Th1 cells from the inflamed joint share T-cell receptor (TCR) clonality with Th17 cells, suggesting a shared clonal origin. They showed that synovial Th17 and Th17/Th1 cells, and unexpectedly, a large proportion of Th1 cells express CD161. Thus, these findings provided evidence that the Th17 phenotype is unstable and that Th17 cells can shift to Th17/Th1 and Th1 cells in human arthritis.

In 2011, Cosmi et al. reported that CD4+CD161+T cells can shift from Th17 cells to the Th17/Th1 or Th1 phenotype in the SF of children with oligoarticular-onset JIA [18]. In addition, they also showed that the accumulation of these cells is correlated with parameters of inflammation. Thus, these findings support the hypothesis that these cells play a pathogenic role in JIA disease activity.

A pathogenic role of the nonclassic Th1 subset has been reported in human inflammation. Nonclassic Th1 is detected in tissue from JIA patients [18,40]. In 2014, Maggi et al. demonstrated that Th17 cells, which express TNFα, can shift to nonclassic Th1 cells in an autocrine or a paracrine manner [9]. In 2013, Maggi et al. reported that CD4+CD161+T cells infiltrate into Crohn’s disease-associated perianal fistulas and that the number of CD4+CD161+ Th cells decreases with anti-TNF therapy, adalimumab [41]. In 2016, they also reported that nonclassic Th1 cells, but not Th17 cells, induce vascular cell adhesion molecule-1 (VCAM-1, CD106) in fibroblast-like synoviocytes from JIA [42]. Ramesh et al. demonstrated that Th17 cells expressing P-glycoprotein (P-gp) produce both Th17 cytokines and Th1 cytokine and that P-gp+ Th17 cells are refractory to the glucocorticoid used to treat clinical autoimmune diseases [43]. Martin-Orozco et al. reported that islet-reactive Th17 cells promote pancreatic inflammation, but only induce diabetes upon conversion into IFNγ producers using non-obese diabetic (NOD) scid mice and neonate NOD mice [44]. Th17 cells-induced diabetes is inhibited by adding anti-IFNγ antibodies, but not anti-IL-17 antibodies in a mouse diabetes model [45]. Thus, it is suggested that nonclassic Th1 cells are more important than Th17 cells per se or Th17/Th1 cells in the pathogenesis of autoimmune diseases.

On the other hand, the pathogenic role of nonclassic Th1 cells in patients with RA remains to be elucidated. In 2014, Miao et al. analyzed the association of circulating Th17 and Th1 cells expressing CD161 with disease activity in RA patients with disease duration of about 4 years under treatment with corticosteroids, disease-modifying anti-rheumatic drugs (DMARDs), or TNF inhibitors [46]. The percentages of CD161+Th17 and CD161+Th1 cells, but not CD161+Th17/Th1 cells, reflected the degree of RA activity. The findings of this study are interesting; however, the various medications possibly have an impact on the findings. Thus, the roles of these cells in the pathogenesis of RA need to be studied using peripheral blood from untreated and early-onset RA patients.

The previous studies of “Th1 cells” in human diseases may need to be evaluated again because “Th1 cells” were divided into classic Th1 cells and nonclassic Th1 [18,19,20,21]. Detection of Th17/Th1 cells [30], CD161 as a marker of human Th17 cells [31], and CD161+Th1 cells as Th17-derived Th1 cells [18] were reported in 2007, 2008, and 2011, respectively. For example, it was reported in 2008 that Th1 cells but not Th17 cells play an important role in the pathogenesis of RA using samples from RA patients whose average disease duration was 13 years [47]. In this study, Th1, Th17, and Th17/Th1 were detected using only the production of cytokines IFNγ, IL-17, and both, respectively, without the detection of CD161. In addition, CD161 plays various roles as mentioned above, except for a role as a marker of human Th17 cells and nonclassic Th1 cells [38,39]. Thus, it is possible that “Th1 cells” included both classic Th1 cells and IL-17-derived nonclassic Th1 cells expressing CD161 in previous studies.

In 2016, we reported elevated ratios of CD161+Th1 cells (i.e., Th17 cell-derived Th1 cells to CD161+Th17 cells) in the peripheral blood of untreated and early-onset RA patients [48]. Recently, it has been reported that Th17 cells can shift to Th1 cells as mentioned above. However, it remained to be elucidated whether this shift occurs in the early phase of RA. In the study, we tried to identify Th17 cells, Th1 cells, and Th17 cell-derived Th1 cells (CD161+Th1 cells) in the peripheral blood of untreated and early-onset RA patients. We identified IL-17(+)IFN-γ(-)CD4(+) T cells as Th17 cells and IL-17(-) IFN-γ(+)CD4(+) T cells as Th1 cells, detecting CD161 on the cell membrane. We also evaluated the effect of methotrexate (MTX) on the ratio of Th17 cells in early-onset RA patients. The ratio of CD161+Th1 cells (i.e., Th17 cell-derived Th1 cells to CD161+Th17 cells) was elevated in the peripheral blood of early-onset RA patients. In addition, MTX reduced the ratio of Th17 cells but not Th1 cells or Th17/Th1 cells. These findings suggest that Th17 cells shift to Th1 cells even in the early phase of RA, and that Th17 cells play important roles in the early phase of RA. In addition, these findings suggest that anti-IL-17 antibodies should be administered to patients with RA in the early phase.

## 4. Th17-Producing IFNγ (IFNγ+ Th17)

The accurate measurement of variations in the human immune system requires precise and standardized assays to distinguish true biological changes from technical artifacts. In 2012, Maecker et al. reported the standardization of cytometry assays and summarized the steps that are required for the Human Immunology Project [15]. In the standardization, the definition of particular subsets of immune cells is conducted using only cell-surface markers without measuring expressed cytokines or CD161.

In 2016, we reported that the ratio of circulating IFNγ+ Th17 cells in memory Th cells is inversely correlated with the titer of anti-CCP antibodies (ACPA) in untreated and early-onset RA patients using flow cytometry methods of the Human Immunology Project [49]. In the same study, we validated the methods of the Human Immunology Project using the cell-surface marker by measuring the actual expressions of IL-17 and IFNγ. In addition, we also evaluated CD161 as a marker of human Th17 cells, measuring the expression of CD161 in human Th17 cells [49].

We tried to identify Th17 cells, IL-17+Th17 cells, and IFNγ+Th17 cells in the peripheral blood of untreated and early-onset RA patients using the method of the Human Immunology Project [49]. Our findings validated the method and the expression of CD161. The ratio of IFNγ+Th17 cells in memory T cells was inversely correlated to the titers of anti-CCP antibodies. It has been reported that cell populations in synovial tissues may shift inversely to those in peripheral blood in RA [13]. Thus, we speculated that IFNγ+Th17 cells are infiltrated in synovial tissue with inflammation in RA patients with high titers of ACPA with decreased peripheral ratio of IFNγ+Th17. These findings suggest that anti-IL-17 antibodies should be administered to patients with early phase RA—especially those with high titers of CCP antibodies.

## 5. Possible Implications for Novel Therapies

Anti-IL-17 antibodies should be administered to early phase RA patients because Th17 cells shift to pathogenic Th17/Th1 or nonclassic Th1, even in the early phase of RA as mentioned above [48,49]. It is speculated that IL-17 is important in the initiation phase of RA, because it has been reported that serum level of IL-17 is higher before the onset of RA than afterward [12]. In phase II trials, anti-IL-17 antibodies, secukinumab or ixekizumab, showed an effect on disease activity of biologics-naïve RA patients or RA patients with an insufficient effect of TNF inhibitor or MTX [50,51,52,53,54,55]. However, phase III trials were closed early because there was no incremental benefit of IL-17 inhibition over other agents currently approved for use in patients who failed TNF inhibitors [56]. We expect that future clinical trials will be performed including only untreated and early-onset RA patients, although this may be difficult to perform.

Interestingly, in 2016, two groups reported the efficacy and safety of monoclonal antibodies targeting the IL-17 pathway for RA using a meta-analysis of randomized controlled clinical trials [57,58]. In addition, in 2017, using meta-analysis, Lee et al. reported associations between circulating IL-17 levels and RA and between IL-17 gene polymorphisms and disease susceptibility [59]. Their meta-analysis revealed significantly higher circulating IL-17 levels in patients with RA, and found evidence of associations between the IL-17A rs2275913, IL-17F rs763780, and IL-17A rs3819024 polymorphisms and pathogenesis of RA.

Interfering with IL-1β, TNFα, IL-23, and IL-12 is important to reduce Th17 cell differentiation into pathogenic Th17/Th1 cells or nonclassic Th1 cells [19,20]. In particular, TNFα is produced by Th17 cells per se. Thus, Th17 cells differentiate to nonclassic Th1 in an autocrine or a paracrine manner [9]. It is speculated that the TNF inhibitors used worldwide reduce the shift of Th17 cells to nonclassic Th1 cells as well as the direct inhibition of TNFα.

In vitro, TNFα and IL-17 show additive or synergistic effects in promoting the production of IL-6, IL-8, and granulocyte-colony stimulating factor (G-CSF), as well as matrix metalloproteinases (MMPs) [60]. Thus, inhibition of both TNFα and IL-17 is potentially attractive for the treatment of RA. Recently, a single antibody (CrossMab_2+2_) combined at the site of Fc binding both IL-17 and TNFα was reported as a therapeutic opportunity in RA using human fibroblast-like synoviocytes (FLS) in vitro and arthritic mice in vivo [60,61]. In addition, another single antibody (ABT-122) binding both IL-17 and TNFα at the site of Fab was reported showing effectiveness and safety in RA therapy [62]. In addition, based on the marketed anti-TNF antibody adalimumab, Silacci et al. generated the bispecific TNF/IL-17-binding FynomAb COVA322 [60]. FynomAbs are fusion proteins of an antibody and a Fyn SH3-derived binding protein. COVA322 is currently being tested in a Phase 1b/2a study in psoriasis [63]. These antibodies may reduce the shift of Th17 cells to nonclassic Th1 cells.

It is important to select RA patients with highly bioactive IL-17 to obtain a sufficient effect of the therapy using anti-IL-17 antibody. Ndongo-Thiam and Miossec constructed a functional bioassay system to measure circulating bioactive IL-17 [64]. Using the system, the IL-17 pro-inflammatory dependent level (IPDL) corresponding to bioactive IL-17 was measured as an anti-IL-17 antibody-inhibition level of IL-8 production from human umbilical vein endothelial cells (HUVEC). The levels were significantly higher in patients with RA versus healthy donors and in destructive versus non-destructive RA, with a positive correlation between IPDL and Larsen score [64]. Thus, this functional bioassay could be used for the selection of RA patients more likely to respond to anti-IL-17 antibody therapy.

Ustekizumab binds p40 (a common molecule of IL-12 and IL-23), and shows the effect of the therapy for psoriasis and psoriatic arthritis [65]. Recently, it has been reported that ustekizumab did not affect the disease activity of RA [66]. However, it is speculated that ustekizumab reduces the shift of Th17 cells to nonclassic Th1 cells.

## 6. Conclusions

Recent studies (including ours) show that the plasticity of Th17 plays important roles in the pathogenesis of RA. However, many issues with the plasticity of Th17 remain to be addressed. Resolution of these issues will provide more strategies for novel therapies.
